# Is Instructional Scaffolding a Better Strategy for Teaching Writing to EFL Learners? A Functional MRI Study in Healthy Young Adults

**DOI:** 10.3390/brainsci11111378

**Published:** 2021-10-21

**Authors:** Hung-Cheng Tai, Chun-Ming Chen, Yuan-Hsiung Tsai, Bih-O Lee, Yulis Setiya Dewi

**Affiliations:** 1 Department of General Education, Chiayi Campus, Chang Gung University of Science and Technology, Chiayi 61363, Taiwan; alextai@gw.cgust.edu.tw; 2 Joint-Appointment Research Fellow, Chiayi Chang Gung Memorial Hospital, Chiayi 613016, Taiwan; 3 Department of Medical Imaging, China Medical University Hospital, Taichung 404332, Taiwan; jinmingc@yahoo.com.hk; 4 Department of Radiology, Chiayi Chang Gung Memorial Hospital, Chiayi 613016, Taiwan; russell.tsai@gmail.com; 5 College of Nursing, Kaohsiung Medical University, Kaohsiung 80708, Taiwan; 6 Center for Medical Education and Humanizing Health Professional Education, Kaohsiung Medical University, Kaohsiung 80708, Taiwan; 7 Faculty of Nursing, Universitas Airlangga, Surabaya 60115, Indonesia; yulis.sd@fkp.unair.ac.id

**Keywords:** brain neural connections, left inferior frontal gyrus, functional magnetic resonance imaging, English as a foreign language, EFL teaching and learning writing

## Abstract

To test the scaffolding theory when applied to the teaching and learning of writing English as a foreign language, this cross-sectional study was conducted to collect physiological data. A total of 53 participants were randomly assigned into two groups, and brain activity was investigated during a guided-writing task using storytelling pictures. The writing task was further divided into four parts using graded levels of difficulty. The experimental group performed tasks in sequence from easy to difficult, whereas the comparison group performed the tasks at random. Outcomes included handwriting assessments and fMRI measurements. Writing outcome assessments were analyzed using SPSS, and scanned images were analyzed using Statistical Parametric Mapping (SPM) software. The results revealed a positive learning effect associated with scaffolding instruction. The experimental group performed better during the writing tasks, and the fMRI images showed less intense and weaker reactions in the language processing region than were observed in the comparison group. The fMRI results also presented the experimental group with reduced motor and cognitive functions when writing in English. This study provides insight regarding brain activity during writing tasks in humans and may have implications for English-language instruction.

## 1. Introduction

Various theories and opinions regarding the most effective methods for teaching writing skills when learning English as a foreign language (EFL) have been proposed, including instructional scaffolding, which is also known as the zone of proximal development (ZPD) [1]. Teachers have adopted this step-by-step strategy to instruct their students, which has become a widely recognized pedagogy worldwide. To examine the effectiveness of these teaching methods, researchers have collected evidence using a behavioral science approach [2]. However, little direct physiological evidence has been reported to support the efficacy of these teaching methods. Whether the human brain is more efficiently activated and trained when using an instructional scaffolding approach compared with other teaching methods remains unclear. Although these issues may have partially been explored in the field of neuroscience, studies have been limited by available technology. Advancements in brain and medical physiological imaging technology have made new research approaches possible. Functional magnetic resonance imaging (fMRI), which is among the most commonly used imaging techniques, detects blood diffusion within the brain by measuring the resonance between the magnetic field and radiofrequency current generated by the machine and the hydrogen atoms (H^+^) contained in the hemoglobin of blood flows to activate brain regions [3].

To examine the effectiveness of applying the instructional scaffolding theory to the process of writing learning in an EFL context, this study used fMRI to measure the brain activities of participants during the performance of a guided-writing task. The topic was presented using storytelling pictures and was further divided into four tasks with various levels of difficulty. The experimental group performed the sequence from easy to difficult (increasing difficulty), whereas the comparison group performed these tasks in a random sequence.

### 1.1. Developing EFL Writing through Scaffolding Instruction

Writing involves complicated interactions between the writer and the target audience. A writer’s prior knowledge and the continuous improvements and revisions made to the writing interact with contextual factors during the writing process. Writing is also a learning activity associated with a considerable cognitive load as indicated by increased brain activity [4]. In many traditional language classrooms, writing is taught by teachers based on the principles of compositional structure and fundamental rhetorical techniques. The topics and questions are provided to students, and the students themselves are responsible for providing the remaining contents. Writing is primarily considered a cognitive activity, and the drafting process is highly sophisticated, involving many levels of brain function. Without sufficient training, novice writers can easily lose direction during the writing process or encounter difficulties, such as writer’s block [5]. For EFL learners, both the complex drafting processes and a lack of linguistic aptitude can increase the “cognitive load” during English-language writing [6]. EFL learners often feel that writing is difficult and become discouraged and uninterested in performing writing tasks [7].

“Cognitive load” refers to the perceptual state of an individual who experiences a mental load or exerts mental effort during the execution of certain activities, assignments, or tasks [8]. Researchers have found that the transition of learners from beginners to professionals relies on the learning process. The most important factor is the transfer of learned concepts from basic ideas into the process of developing problem-solving strategies and models [8]. However, information that is irrelevant to the learning process, such as emotions, doubts, and frustrations, can occupy short-term memory and increase cognitive load [6]. This hypothesis suggests that pedagogies and teaching approaches that require lower cognitive loads can improve learning. Researchers committed to studying cognitive loads experienced by learners during different teaching methods and when exposed to different content have established the cognitive load theory [9].

Among these teaching approaches, the notion of scaffolding or ZPD, which was first proposed by Vygotsky (1987) [10], is among the most effective strategies. Teachers at various levels and across professional domains have adopted this step-by-step strategy to instruct their students [1,11]. Similarly, in ancient Chinese education, the original meaning of the proverb “循序漸進” is to arrange the parts of a written composition first according to the arrangement of the article segments, followed by the selection and organization of the proper words and sentences. The proverb was later developed to refer to learning that follows certain rules and procedures. The scaffolding approach has inspired language teachers to develop the guided-writing approach to ease the level of task difficulty for novice EFL writers [1]. A common method for teaching guided writing is the presentation of simple pictures as the writing topic to help the writers visualize the content and structure of the writing task [7]. When the task content is relevant to the writers’ living experiences, the cognitive load generated by creativity and abstract thinking can be reduced. Writers instead only need to focus on the construction, semantics, and syntax of the target language. Advocates believe that this strategy can improve the writing outcomes for EFL learners and enhance their learning motivation [12].

### 1.2. Relationship between Brain Activation and Language Processing

Early studies applying fMRI technology to the study of language processing initially focused on individual words and gradually advanced to the sentence level [13]. Recent studies show that reading and understanding sentences involve approximately 10 different brain regions [13], and include their subcortical structures, too. The comprehensive language network also goes considerably beyond these ten areas [14]. Although each study involved a different research purpose and design, the main activation areas were frequently concentrated in the left inferior frontal gyrus (left IFG), particularly in the left pars opercularis (left POp), the precentral gyrus (PG), and the pars triangularis (PTr) [15,16,17]. In addition, studies have shown the left middle temporal gyrus (left MTG) and the left superior temporal gyrus (left STG) are also important for language processing [17,18,19]. A recent study suggests the posterior superior temporal sulcus (STS) [20] should be included as well.

Further distinctions between the two main functions of language processing, semantics and syntax, are separately discussed. The areas where semantics are processed include the regions of the left IFG known as the Brodmann areas, BA44 and BA45 [21]. Other areas include the left STG [22] and left inferior temporal gyrus (ITG) [23]. The left pars orbitalis (POrb) [24] has been found to be significantly activated. Syntax processing appears to be more dispersed, and at least 7 areas have been proposed: BA44/left IFG [25]; left insula [17,19,26]; precuneus [15,24]; left MTG [18,19,27]; left PG [28]; supplementary motor area (SMA) [17,29]; and left supramarginal gyrus (left SMG) [24].

The literature exploring the brain activation associated with language processing at the sentence level has accumulated over the past decade, providing linguists and psychologists with increasing insight regarding the major brain regions involved in processing semantics and syntax. However, the higher-order and more complex brain functions that are activated during writing processes have rarely been studied and appear to be worth further exploration.

### 1.3. Relationship between Brain Activation and Writing Activities

A total of 13 brain areas can be categorized as directly involved in writing, motion control, and language in the cortex and sub-cortex. The 5 areas identified as directly associated with writing include the left superior frontal gyrus (left SFG), middle frontal gyrus (MiFG), left intraparietal sulcus (IPS), superior parietal lobe (SPL), and right cerebellum [30]. Motion-related areas include the primary motor cortex, sensorimotor cortex, SMA, thalamus, and putamen. Language-relevant functions activate the ventral premotor cortex and posterior and inferior temporal cortex. These functional areas plus areas involved in visual processing constitute the reference areas referred to by the current study [30].

Karimpoor et al., 2018 [3] used a touchscreen tablet to simulate a handwriting experiment performed while the subjects were monitored in an fMRI machine, and the results showed that the activated brain areas were similar to those identified during previous pencil and paper research. Motor-related brain regions included the left-lateralized primary somatosensory cortex, motor cortex, bilateral SMA, and premotor area. Visual functions activate the primary visual and visual association areas. The areas related to writing and language include the SPL, IPG, MiFG/SFG, STG, Wernicke’s area, posterior cerebellum (postCB), angular gyrus (ANG), supramarginal gyrus (SuMG), bilateral IFG/Broca’s area, and ventral premotor cortex on both sides of the brain. Another crucial writing area, named the visual word form area (VWFA), is located in the inferior temporal sulcus (ITS)/middle occipital gyrus (MiOG) of the left hemisphere [3].

Shah et al. [31] attempted to understand how the brain works during the process of creative writing. In their research design, native German writers in the control group copied contents directly after reading an article without requiring creativity or innovation. During simple writing activities, such as transcription or copying, areas that include the PG, bilateral dorsal premotor cortex (dPMC), left SMA, and right anterior cerebellum show stronger activation, based on the level of blood perfusion. Moreover, bilateral activation can be detected in the dorsolateral prefrontal cortex (DLPFC or BA 46/9) and the insula. Other areas are activated unilaterally, including the left Rolandic operculum (ROL) and the right IFG. In addition, the bilateral visual cortex, left thalamus, left putamen, and other areas also showed strong activation signals [31].

### 1.4. Study Aims

The two primary purposes of this study include validating whether the scaffolding approach, represented by the sequence of writing tasks, affected the participants’ (a) writing outcomes and (b) brain activation when comparing between two groups.

We hypothesized that the level of brain activation would be proportional to the cognitive load, with more activated brain regions and higher levels of activation, indicating heavier perceived cognitive loads.

## 2. Materials and Methods

### 2.1. Research Design

This study used a cross-sectional, mixed block and event-related experimental design with two groups. The difference between the two groups was the sequence in which the same four writing tasks were presented. The four tasks were transcribing in Chinese (Task 1), translating Chinese into English (Task 2), writing in Chinese (Task 3), and writing in English (Task 4). The experimental group performed these four tasks in the presented order, which progresses from easier to more difficult tasks. The comparison group performed these tasks in random order. The independent variable was the order in which the writing tasks were performed, whereas the dependent variables were the writing outcomes, activated brain activation areas, and the levels of brain activations.

### 2.2. Participants

A total of 53 participants were assigned randomly to the two groups, resulting in 27 participants in the experimental group and 26 participants in the comparison group. Their ages ranged from 21 to 28 years (Mean: 22.45 years; standard deviation [S]: 1.60 years) and included 40 women and 13 men (5 in the experimental group and 8 in the comparison group), who were primarily recruited from a nursing university. Right-handed individuals were preferred to ensure similar brain activity patterns. Normal vision or corrected-to-normal vision using contact lenses was required to ensure that the words and pictures could be clearly viewed while in the fMRI machine. Participants were included if they were healthy, without any neurodegenerative, mental, or cognitive disorders, and had never experienced severe brain injury or undergone any form of brain surgery. Pregnant women and those with contraindications preventing fMRI machine examinations, such as cardiac rhythmic devices, were excluded.

All participants were native Chinese speakers with various levels of EFL experience. Most participants were nursing students studying in the two-year or four-year technical program, either in the day (*n* = 28) or night school (*n* = 18). The remaining 7 participants were employers working in the healthcare industry.

Their language competencies were diverse, ranging from A1 to B2 (Common European Framework of Reference for Language, CEFR). To investigate the initial differences of EFL language competence among the two groups, participants were assessed using a 40-item reading examination at approximately the General English Proficiency Test (GEPT) [32] intermediate level or CEFR B2. This reading examination had been previously tested to have satisfactory validity and reliability. Each item on this multiple-choice examination was worth 2.5 points, resulting in a maximum possible score of 100. The assessment results showed the mean scores were 56.85 ± 15.07 (experimental) and 49.62 ± 14.08 (comparison group). A univariate test showed no significant difference between the two groups (*F*(1, 51) = 3.26, *p* = 0.077).

### 2.3. Writing Tasks

**Figure 1:**
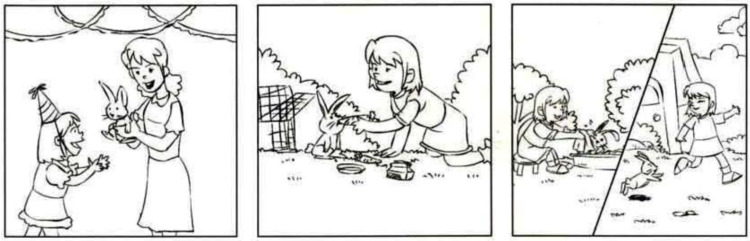
Figures of the storytelling composition.

The writing tasks were picture-guided paragraphs derived from a GEPT at the elementary level or CEFR A2 equivalent (see Figure 1). After entering the fMRI machine, participants were instructed to write on the topic, guided by a set of pictures.

**Figure 2:**
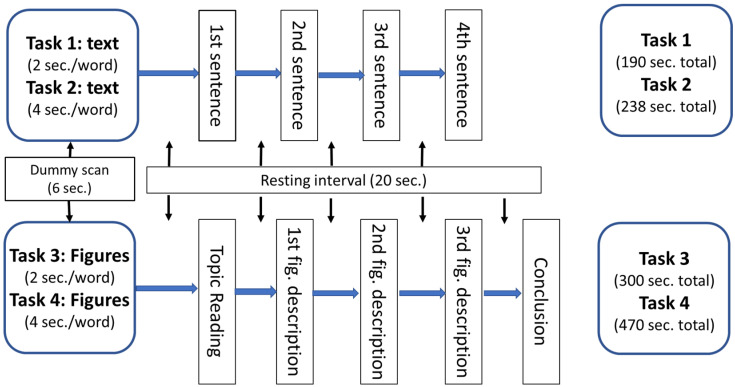
Protocol of the experiment.

The writing tasks were divided into multiple steps to allow for the fMRI to record brain activation patterns in detail (see Figure 2). The four tasks were designed with different levels of difficulty (see Appendix A). The first task asked the participants to transcribe a few sentences that briefly outlined the topic using their native language of Mandarin Chinese. The second task asked the participants to translate the first task into English. The third task was to draft a full story on this topic using Chinese, which was considered more advanced than the second task. The fourth task was to draft a full story on this topic directly in English, which was considered to be the most difficult. Based on Vygotsky’s scaffolding theory, performing this sequence of tasks in the order 1–2–3–4, moving from easy to difficult, should be the conventional writing instruction method for the intervention used in the experimental group.

### 2.4. Data Collection

#### 2.4.1. Procedures

Before fMRI scanning, all participants were fully informed regarding the research purposes and procedures by the research assistants. Their EFL language competence was assessed through a reading examination, and all participants received standardized writing training before undergoing functional scans to assure that they understood the task performance procedures. The goal of the training was to familiarize the participants with the length and difficulty of the writing tasks and procedures. Participants were provided with details regarding the functional scans, including the expected durations, writing task formats, language use, and the anticipated experience of being inside the machine. Three instruments were used to collect the data: two to assess language abilities and one to acquire brain images.

#### 2.4.2. Instruments

- Instrument 1: Writing outcomes

The writing outcome of each participant was assessed using established criteria (see Table 1). The “readability” criterion refers to the quality and recognition of the handwriting produced while in the fMRI machine. Speed refers to the degree to which each participant completed each task. The other 5 criteria, including content, structure, grammar, vocabulary, and punctuation, are commonly used in language tests nationwide [33]. The overall score was obtained by averaging the scores for all measured criteria. To ensure the consistency and reliability of these assessments, an experienced EFL teacher was invited to assist in the writing task assessments.

- Instrument 2: fMRI images

This functional experiment used the SIEMENS Verio 3T MRI scanner located in Chiayi Chang Gung Memorial Hospital. The total scanning time was approximately one hour, including an additional scan for magnetization-prepared rapid acquisition with gradient echo (MPRAGE, 7 min). The protocol used for the experiment is shown in Figure 2.

The MPRAGE acquisition required participants to look at a cross mark (+) on the screen with no other activity. A set of high-resolution structural images (T1 3D spoiled gradient-recalled [SPGR]/MPRAGE) were obtained to locate brain areas as functional result markers. The main scan collected information about the activation of brain regions during the four writing tasks. Two devices, a handwriting desk and a projection screen made from acrylic material, were installed (see Appendix B), which allowed the participants to read the topic and perform handwriting while inside the machine.

During the scanning process, the participants’ heads were constrained within a reasonable range because fMRI is sensitive to body movements. We adopted two strategies for fixing the positions of a participant’s head: we fit sponges around the head within the frame of the fMRI machine and limited each round of tests to eight minutes, separated by rest intervals.

### 2.5. Data Analysis

Two types of data analyses were performed. To assess the writing outcomes, a multivariate analysis of variance (MANOVA) was conducted using SPSS 21.0 [34]. All 27 writing outcome criteria were set as dependent variables and the group (experiment vs. comparison) as fixed variable. To further identify the exact dependent variables contributed to the significance of the MANOVA, pairwise comparisons and univariate testing for the factor of “group” were performed, along with their corresponding effect sizes of Cohen’s d and r. As the MANOVA test integrated all variables into one model, all relationships between the variables were calculated simultaneously. Type 1 errors adjustment is often seen in the multiple tests of single relationships, and could be controlled and avoided in the MANOVA.

The acquired fMRI images were digitized using Statistical Parametric Mapping (SPM 12.0) [35] software. Data pre-processing consisted of four basic steps: slice-timing correction; motion correction; co-registration and spatial normalization; and smoothing. The data processing stage was performed in 4 steps: design metrics, import data, stepwise analysis, and type-one error consideration.

A generalized linear model (GLM) analysis was adopted to statistically analyze the digitized data. The first level of analysis consolidated an individual’s brain images, and the second level compared the data within or between groups. The completed data were then presented in SPM and xjView for a more dynamic display. To calculate the effect size of the test, we followed the method proposed by Lombardo et al. [36].

## 3. Results

### 3.1. Writing Outcomes

The MANOVA results revealed a significant difference in the writing outcomes between the two groups [*F*(27, 25) = 4.45, *p* < 0.001]. Through using the software of G*Power 3.1 [37], effect sizes of this MANOVA were obtained: f^2^(V) = 1.15 and Pilai V = 0.53. The huge effect sizes indicated strong relationships existed between dependent variables among the two groups.

Table 2 demonstrates the univariate effects for “group” of the MANOVA. A total of 10 assessment criteria (dependent variables), including “content” and “structure” of the Task 2, 3, 4, and “speed”, “grammar”, “vocabulary”, and “holistic” of the Task 3, were identified. They all showed strong effect sizes among the groups, too.

Furthermore, to inspect whether the initially language-competence differences among the two groups would influence the MANOVA results, additional tests of multivariate analysis of covariance (MANCOVA) was performed. Participant’s initial language competence was set as a covariate, and both tests showed highly consistent results (see Appendix C).

### 3.2. Brain Images

To further analyze the brain images acquired during the 4 writing tasks, the various actions performed during the writing processes were separated into 8 segments (Figure 2): transcription in Chinese, translation in English, topic and figure comprehension in Tasks 3 and 4, figure description in Chinese and English, and conclusion writing in Chinese and English. Comparisons between the experimental and comparison group revealed differences in the activation patterns for each segment.

#### 3.2.1. The Main Effect of the Handwriting Tasks

**Figure 3:**
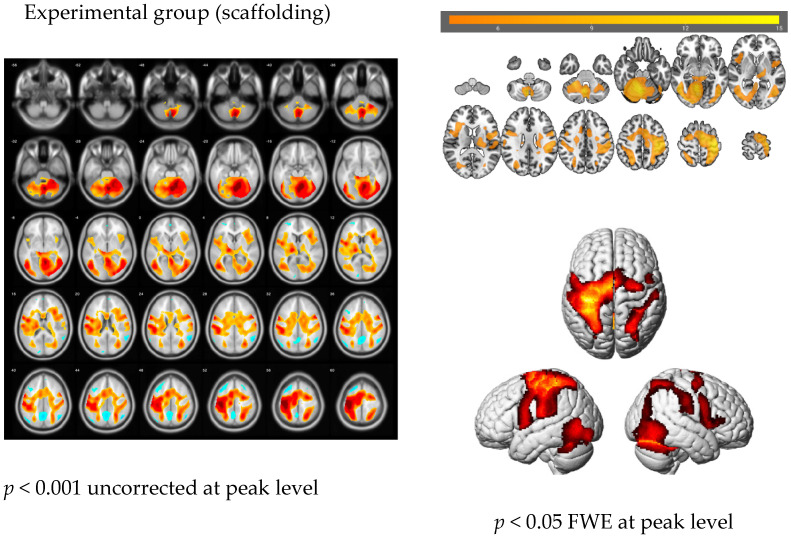
Handwriting Task 1: Transcription in Chinese.

Although the 8 task segments were analyzed, only one is shown to provide a representative figure. The remaining 15 figures can be provided upon request. Figure 3 (left) demonstrates the brain activation patterns associated with Task 1 (transcription in Chinese) for the experimental group. The global peak of activity was identified in the cerebellum (x, y, z = 6, −62, −20; T = 14.48; cluster = 65,332; *p* < 0.001 uncorrected peak level), cover a huge cluster containing many different regions. The value of Cohen’s d of this condition is 4.12, implying a huge effect size. To identify the most significantly activated regions, a further analysis was performed, setting the threshold as *p* < 0.05 family-wise error (FWE)-corrected at the peak level, as shown in Figure 3 (right) and Table 3.

#### 3.2.2. The Language Effects among Different Tasks

**Figure 4:**
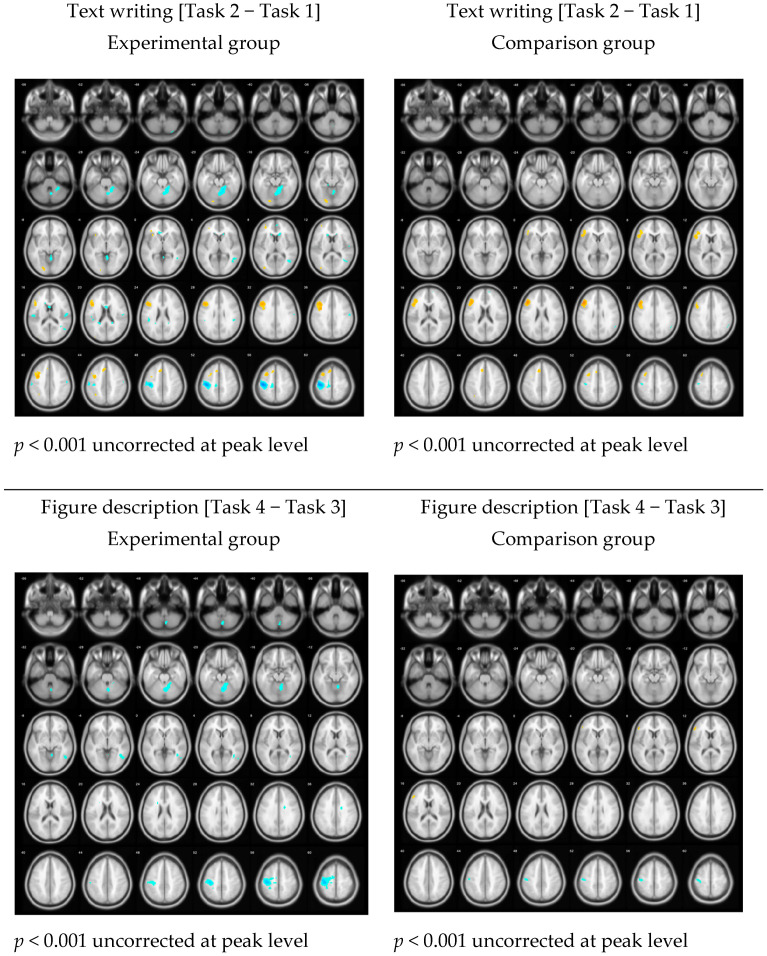
The language effect among different tasks.

Four findings illustrate the language effects among the tasks between the two groups. First, the experimental group had shown lower activation when translating Chinese into English [(English < Chinese); (Task 2) < (Task 1)]. Figure 4 shows the experimental group had negative activation in the motor-related areas. The left postcentral (x, y, z = −40, −22, 56; T = 7.04; cluster = 1544; *p* < 0.05 FWE cluster level), and right cerebellum (x, y, z = 6, −62, −20; T = 4.87; cluster = 1158; *p* < 0.05 FWE cluster level) were identified as different between the two groups but the difference was not significant.

Second, the comparison group had stronger activation than the experimental group in the left triangular region of the IFG (x, y, z = −46, 26, 28; T = 5.09; cluster = 1280; *p* < 0.05 FWE cluster level). The experimental group had significant activation in the left PG (x, y, z = −38, 4, 40; T = 4.67; cluster = 1235; *p* < 0.05 FWE cluster level), consisting of the left IFG opercula (BA9) (x, y, z = −38, 20, 34; T = 4.53) and left triangular IFG (BA46) (x, y, z = −46, 20, 28; T = 4.38), which are relevant to language processing.

Third, the experimental group had lower activation when drafting storytelling in English [Task 4 < Task 3]. For the experimental group, their brain activation was not strong in the language-related areas but were strong in the motor functional areas (English < Chinese): left PG (x, y, z = −36, −22, 62; T = 5.68; cluster = 1793; *p* < 0.05 FWE cluster level) and right cerebellum (x, y, z = 6, −62, −22; T = 4.68; cluster = 694; *p* < 0.05 FWE cluster level). However, no significant differences between English and Chinese drafting were observed for the comparison group during the drafting tasks.

Fourth, neither group showed significant differences when writing conclusions. Generally, the two groups did not show clear activation differences in any brain areas.

As to the effect sizes of the four conditions, Cohen’s d values for the experimental group are 1.80 (Text writing [Task 2 − Task 1]) and 0.96 (Figure description [Task 4 − Task 3]). For the comparison group are 1.43 and 0.93, respectively.

#### 3.2.3. Learning Effects through Scaffolding Instruction

**Figure 5:**
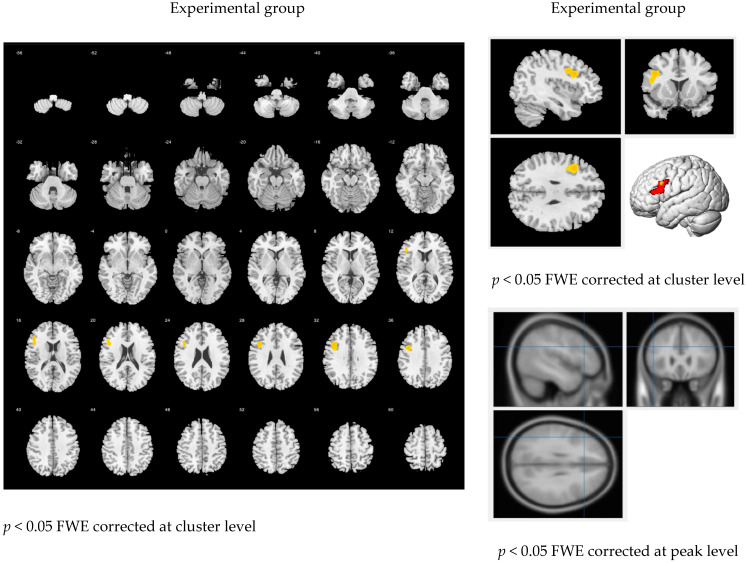

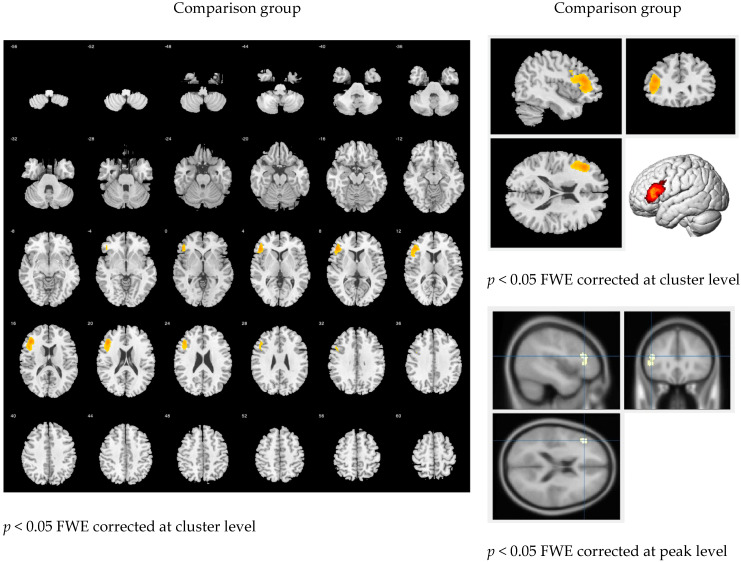
Learning effect between two groups: [(Task 2 + Task 4) − (Task 3 + Task 1)].

Figure 5 demonstrates the consolidating of all 4 writing tasks within each group. They show similar patterns during writing (left figures). The local peak identified in the experimental group was observed in the left IFG (x, y, z = −40, 14, 30; T-value = 3.93; cluster size = 565; *p* < 0.05 FWE cluster level). A similar area was also observed in the comparison group (x, y, z = −44, 30, 18; T-value = 5.13; cluster size = 1154; *p* < 0.05 FWE cluster level). Effect sizes of the two conditions are Cohen’s d = 3.03 (experimental) and Cohen’s d = 2.75 (comparison).

However, after adjusting the threshold of significance to a stricter *p* < 0.05, FWE-corrected at the peak level, a difference between the two groups appeared. The experimental group showed no significant activation in the left IFG (*p* = 0.265), whereas the comparison group remained significant (*p* = 0.002).

Moreover, both groups had significant negative activation [English < Chinese] under this condition. The experimental group showed activation in the left PG (x, y, z = −38, −22, 58; T = 6.60; cluster = 1824; *p* < 0.05 FWE cluster level) and right caudate (x, y, z = 16, 28, 6; T = 4.89; cluster = 886; *p* < 0.05 FWE cluster level). Similarly, the comparison group showed significant activation in the left PG (x, y, z = −32, −28, 66; T = 4.55; cluster = 1048; *p* < 0.05 FWE cluster level).

## 4. Discussion

The significance of this study is the application of fMRI in an educational context to understand the brain activation associated with a learning activity. The results of both the behavior measurement and the fMRI analysis demonstrated significant differences between the two groups. The learning effect observed on the experimental group’s writing outcomes and brain activation patterns provided a complete picture of the effects of scaffolding instruction.

### 4.1. Writing Outcomes

The behavioral measurement results indicated that the scaffolding approach was effective in assisting the process of learning the content and structure dimensions during the writing tasks. The experimental group performed better than the comparison group in Tasks 2, 3, 4, but no difference was observed for Task 1 (transcription in Chinese); as Task 1 was the easiest task, no significant difference was observed, regardless of when it appeared in the task sequence. Of the 27 evaluation criteria, those associated with content and structure showed significant differences in all 3 tasks in which they were measured, which suggested that the scaffolding approach effectively assisted participants in the dimensions of content formation and structure construction and indicating that the cognitive load [6] of the experimental group was reduced during story drafting using either language.

Task 3 (drafting in Chinese) benefited the most from scaffolding instruction. Six dimensions, including the holistic outcome, showed significant differences between groups. Although the participants used their native language, Chinese, the step-by-step scaffolding supplied the experimental group with a starting point and a genre to guide the direction of the writing task. They were less likely to experience the status of mind blanking, or writer’s block, which are frequently reported in the writing and neural literature [38]. During Tasks 2 (translation) and 4 (draft), which involved English, the knowledge of linguistic forms, such as EFL vocabulary and grammar, might not be assisted through any means of instruction.

### 4.2. Brain Images

First, the main effects observed during the handwriting tasks demonstrated consistency with previous literature. Although only one of the 8 conditions was displayed, the typical activation of the brain network associated with handwriting was illustrated. To compare the current studies with previous studies, four network functions were categorized: writing directly, language processing, motor functions, and visual functions. The categorizations were roughly defined, as most of the regions are multifunctional and involved in various tasks. The writing process involves the cerebellum [30,31], SFG [3,30], midcingulate cortex (MCC), superior parietal gyrus [3,30], and fusiform gyrus (VWFA) [3]. Language processing functions are associated with the IFG [24,31], Broca’s area [3,26], POp [16], inferior parietal gyrus (IPG) [3,24], MTG [17,18,19], ROL [31], and ANG [3]. Further distinctions have been recognized between the processing of semantics, which involves the ITG [23,30], and syntax, which occurs in the SMG [24] and insula [17,19,26,31]. Motor functions were associated with activity in the primary motor cortex [15,16,17,30,31], primary somatosensory cortex [3], SMA [3,17,30,31], and thalamus [30,31]. The MiOG [3] and inferior occipital gyrus [4] are activated during visual functions. The appearance of activity in these areas can be used to confirm the validity and reliability of this experiment. However, the MCC is not frequently mentioned in the language field. According to Hoffstaedter et al. (2014), the MCC is involved in the cognitive control of movement generation or intentional motor control [39], and the specific function of the MCC during the writing process may require further investigation.

Second, when examining the language effects across the different tasks, the experimental group showed lower levels of brain activation when translating Chinese text into English compared with the comparison group. This may be associated with the stronger activation observed for the experimental group in the motor-related areas, such as the left postcentral and right cerebellum, compared with the comparison group during the Chinese transcription task. This phenomenon might be relevant to the adjustment to the weird postures lied inside the fMRI machine during the first task among the experimental-group participants. After they became familiar with the context, they might have become more efficient and comfortable during the later tasks. By contrast, no similar adjustment was observed for the comparison group because the task sequence was presented randomly, preventing the identification of a clear learning curve associated with any specific task. When examining the individual conditions, the comparison group showed stronger activation during the comprehension of topic figures and reading phases within each task. As the comparison group had the opportunity to see these topic figures as their first task, they did not display the efficient and relaxed status observed for the experimental group and instead displayed higher activations during each task. The randomized order of presentation might increase the risk-taking opportunities, the level of difficulty, and the cognitive load for the comparison group during each task than the experimental group.

The comparison group showed stronger activation in the left IFG triangular area (Figure 4), which has been consistently demonstrated to be relevant to language processing [18]. This result suggested the comparison group consumed more energy when translating texts from Chinese to English. Although similar areas (the left IFG opercula and triangular area) were activated in the experimental group, the level of activation was weaker than in the comparison group. As this task involved the simple transfer of information between two languages, these results have interesting implications for EFL acquisition. The learning effect observed that was observed in the experimental group was not as apparent in the comparison group. The language functions that were operational to perform the transfer between these two languages were more obvious in the comparison group than in the experimental group. This finding indicated that the comparison group concentrated more strongly on language control when shifting from one language to another.

The experimental group displayed reduced activation when drafting during the storytelling task in English. Figure 5 demonstrates the differences between Tasks 3 (drafting in Chinese) and 4 (drafting in English). For the experimental group, brain activations were not strong in the language-related areas, but significantly increased activation was observed in the cerebellum and motor areas compared with the comparison group. The negative activation of the left PG may be associated with the logographic property of Chinese compared with the English alphabetic system, resulting in Chinese characters requiring more sophisticated motions and coordination than the English alphabet.

During the writing of the conclusion, no significant difference was observed between groups, which might indicate that conclusion writing does not require significant brain resources for participants. They were able to retrieve previous wording and then generate a one-sentence summary.

Third, the results of the learning effect showed the experimental group presented with significantly reduced levels of brain activation in the language area (i.e., the left IFG) than the comparison group. The sequential task order used in the experimental group may have provided a learning effect that assisted with the performance of these tasks in a smooth and consistent manner. The scaffolding approach assisted the experimental group with the drafting process and reduced their cognitive loads during language processing tasks. By contrast, the presentation of tasks in a randomized order might increase the difficulty of the writing process, as the comparison group was unable to predict subsequent tasks. The added difficulty associated with this unpredictability might induce increased cognitive load, triggering an increase in the level of blood infusion into the left IFG area to help solve problems.

Lastly, the experimental group showed stronger activation in two areas, the left PG and right caudate, during Chinese writing compared with English. The experimental group had stronger activation in the left PG, a motor area known to be associated with the movement of the right hand. The scaffolding approach provided practice during the first task in Chinese, which may have made the subsequent task performed in English easier for the experimental group. The right caudate serves as an important node during the acquisition of second languages and controls the processes during multilingual or polyglottal activities [40]. The activation of the right caudate indicated that the experimental group experienced better control of switching between languages.

### 4.3. Implications and Limitations

The results of this study lead to three implications. For educational settings, the introduction of fMRI technology can supply physiological evidence to verify the effectiveness of teaching and learning techniques, in addition to evaluating traditional behavioral measurements. In the EFL learning context, studying the language switching effect can be used to provide a deeper understanding of how the human brain works when transitioning between languages. For healthcare professionals, the pattern of brain activation measured in healthy adults can be used for later comparisons. Understanding efficient practices for teaching writing may facilitate the training and diagnosis of individuals with dysfunctional language processing. Finally, although the scaffolding approach has been widely recognized, scaffolding is not a panacea that is suitable for every context. For those teachers who would like to provide additional challenges or design a flipped classroom as a pedagogical approach, scaffolding represents only one of several options.

Four limitations are proposed for the current study: First, the teaching and researching context was a nursing university, and most of the participants in this study were women. Although we attempted to balance the gender ratio, we encountered difficulty and limitations during recruitment. Second, the fMRI machine was in a teaching hospital and was installed for clinical diagnosis, not designated for research. The machine availability and reliability were out of our control. Third, because the fMRI machine was in an isolated and restricted space, free from magnetic objects, tracking devices, such as video cameras, eye-tracking technology, and tablets were difficult to incorporate. We were not able to develop a mechanism for the detailed monitoring of the writing process during task performance. The exact times when they started, paused, or stopped writing were unknown. Fourth, the assessment criteria for the writing outcomes were implemented long before the introduction of fMRI technology, and the discrepancy between the two measurements made them incomparable to some findings and results.

## 5. Conclusions

This study was an experimental comparison between two groups of young, healthy adults who performed four handwritten writing tasks using two languages while being monitored inside of an fMRI machine. The behavior results showed the experimental group performed better during drafting tasks than the comparison group, especially during Task 3 (drafting in Chinese). The fMRI results revealed the brain activation patterns observed in this study corresponded with those identified during previous brain research, confirming the validity and reliability of our study. The experimental group also demonstrated significantly reduced activation in the language areas (i.e., the left IFG) than the comparison group. The guided-writing approach might have helped the participants scaffold their drafting process, reducing the cognitive loads associated with the language processing functions.

The experimental group also showed reduced brain activation levels during English translation and writing than the comparison group. The step-by-step instruction might reduce the participants’ cognitive load when controlling the switch to the English language. The fMRI research provides physical evidence to support the behaviorists’ viewpoint that scaffolding represents a valuable teaching strategy for the teaching and learning of EFL writing.

## Figures and Tables

**Table 1:** Outcome measure criteria for the four writing tasks.

Task		Criterion 1	2	3	4	5	6	7	8
1	Transcribe in Chinese	readability	speed	-	-	-	-	-	holistic
2	Translate in English	readability	speed	content	structure	grammar	vocabulary	punctuation	holistic
3	Draft in Chinese	readability	speed	content	structure	grammar	vocabulary	punctuation	holistic
4	Draft in English	readability	speed	content	structure	grammar	vocabulary	punctuation	holistic

The 0–5 grading standards of the writing tasks were: 0—unanswered or equivalent. 1—only little part answers to the question, and seriously affects the reader’s comprehension. 2—partly answer to the question, and readers had to read carefully to comprehend. 3—mostly answer to the question and the reader can comprehend without much difficulty, but still have some grammar and vo-cabulary mistakes. 4—clearly answer to the question and the reader can comprehend easily with minor grammar or vocabulary mistakes. 5—clearly answer to the question and the reader can comprehend easily, and there is almost no grammar or vocabulary mistake.

**Table 2:** Univariate effects for “Group” of the MANOVA test.

Dependent Variables		df/ df Error	*F*	Group	Means	99.9% Confidence Interval		Cohen’s d	Effect Size r
						Lower	Upper		
Task 1	Readability	1	0.08	Experiment	4.00	3.35	4.65	0.08	0.04
		51		Comparison	4.08	3.41	4.74		
	Speed	1	0.06	Experiment	3.96	3.19	4.74	0.07	0.03
		51		Comparison	4.04	3.25	4.83		
	Holistic	1	0.61	Experiment	4.04	3.43	4.64	0.21	0.10
		51		Comparison	4.23	3.61	4.85		
Task 2	Readability	1	1.78	Experiment	3.93	3.29	4.57	0.37	0.18
		51		Comparison	3.58	2.93	4.23		
	Speed	1	1.89	Experiment	4.04	3.42	4.65	0.38	0.19
		51		Comparison	3.69	3.07	4.32		
	Content	1	24.79 **	Experiment	3.78	3.21	4.35	1.37	0.57
		51		Comparison	2.62	2.03	3.20		
	Structure	1	17.23 **	Experiment	3.70	3.15	4.25	1.14	0.50
		51		Comparison	2.77	2.21	3.33		
	Grammar	1	4.84	Experiment	3.00	2.40	3.60	0.61	0.29
		51		Comparison	2.46	1.85	3.07		
	Vocabulary	1	13.68	Experiment	3.26	2.63	3.90	1.02	0.45
		51		Comparison	2.31	1.67	2.95		
	Punctuation	1	1.10	Experiment	3.59	2.93	4.26	0.28	0.14
		51		Comparison	3.31	2.63	3.99		
	Holistic	1	6.38	Experiment	3.52	2.95	4.09	0.70	0.33
		51		Comparison	2.93	2.35	3.51		
Task 3	Readability	1	0.93	Experiment	3.93	3.24	4.62	0.27	0.14
		51		Comparison	3.65	2.95	4.36		
	Speed	1	22.69 **	Experiment	4.04	3.41	4.67	1.31	0.55
		51		Comparison	2.81	2.16	3.45		
	Content	1	22.90 **	Experiment	3.93	3.28	4.58	1.33	0.55
		51		Comparison	2.65	1.99	3.32		
	Structure	1	22.79 **	Experiment	4.07	3.41	4.74	1.31	0.55
		51		Comparison	2.77	2.09	3.45		
	Grammar	1	26.64 **	Experiment	4.19	3.53	4.84	1.42	0.58
		51		Comparison	2.81	2.14	3.47		
	Vocabulary	1	43.90 **	Experiment	4.15	3.55	4.74	1.83	0.67
		51		Comparison	2.54	1.93	3.14		
	Punctuation	1	9.48	Experiment	4.22	3.65	4.80	.85	0.39
		51		Comparison	3.50	2.92	4.09		
	Holistic	1	24.40 **	Experiment	4.11	3.54	4.68	1.36	0.56
		51		Comparison	2.96	2.37	3.54		
Task 4	Readability	1	3.71	Experiment	3.67	3.11	4.22	0.53	0.26
		51		Comparison	3.23	2.67	3.80		
	Speed	1	12.86	Experiment	3.93	3.37	4.48	0.98	0.44
		51		Comparison	3.12	2.55	3.68		
	Content	1	21.43 **	Experiment	3.48	2.88	4.08	1.27	0.54
		51		Comparison	2.35	1.74	2.96		
	Structure	1	14.75 **	Experiment	3.41	2.81	4.01	1.06	0.47
		51		Comparison	2.46	1.85	3.08		
	Grammar	1	4.19	Experiment	2.89	2.33	3.45	0.57	0.27
		51		Comparison	2.42	1.86	2.99		
	Vocabulary	1	11.72	Experiment	3.22	2.57	3.88	0.94	0.43
		51		Comparison	2.31	1.64	2.97		
	Punctuation	1	3.09	Experiment	3.48	2.81	4.15	0.48	0.23
		51		Comparison	3.00	2.32	3.68		
	Holistic	1	8.02	Experiment	3.37	2.79	3.95	0.78	0.36
		51		Comparison	2.70	2.11	3.29		

Note: ** *p* < 0.001.

**Table 3:** Experimental-group outcomes for Task 1: Transcription in Chinese.

Structure	Anatomy	Abbreviation	Hemisphere	x, y, z	T-Value	Cluster Size
Lateralized Regions						
Precentral_L	Primary Motor Cortex	M1	L	−40, −24, 58	14.16	2074
Postcentral_L	Primary Somatosensory Cortex	S1	L	−36, −36, 58	11.45	2465
Supp_Motor_Area_L	Supplementary Motor Area	SMA	L	−8, −8, 64	10.01	1412
SupraMarginal_L	Supramarginal Gyrus	SMG	L	−44, −30, 24	8.19	599
Thal_VPL_L	Thalamus	THA	L	−16, −20, 8	8.28	158
Angular_R	Angular Gyrus	ANG	R	30, −58, 44	6.03	96
Bilateral Regions						
Frontal_Sup_R	Superior Frontal Gyrus	SFG	R	26, −4, 54	7.68	495
Frontal_Sup_L			L	−24, −8, 54	8.95	527
Frontal_Inf_Oper_R	Inferior Frontal Gyrus	IFG	R	54, 8, 26	7.51	366
Frontal_Inf_Oper_L	(pars opercularis)	Broca’s area	L	−54, 6, 14	6.47	160
Rolandic_Oper_R	Rolandic operculum	ROL	R	40, 2, 14	5.88	148
Rolandic_Oper_L			L	−44, −2, 14	7.97	616
Insula_R	Insula	INS	R	34, 20, 12	7.47	470
Insula_L			L	−32, 18, 12	5.17	280
Cingulate_Mid_R	Midcingulate Cortex	MCC	R	10, 0, 34	5.5	249
Cingulate_Mid_L			L	−10, 0, 36	5.55	363
Parietal_Sup_R	Superior Parietal Gyrus	SPG	R	24, −60, 54	8.2	685
Parietal_Sup_L			L	−26, −60, 62	8.44	1103
Parietal_Inf_R	Inferior Parietal Gyrus	IPG	R	36, −44, 50	5.55	246
Parietal_Inf_L			L	−38, −42, 44	6.28	994
Temporal_Mid_R	Middle Temporal Gyrus	MTG	R	50, −60, −2	6.98	285
Temporal_Mid_L			L	−46, −66, 8	6.25	388
Temporal_Inf_R	Inferior Temporal Gyrus	ITG	R	50, −58, −12	9.95	422
Temporal_Inf_L			L	−46, −54, −14	6.83	227
Occipital_Mid_R	Middle Occipital Gyrus	MOG	R	34, −72, 24	6.45	493
Occipital_Mid_L			L	−44, −68, 0	7.84	524
Occipital_Inf_R	Inferior Occipital Gyrus	IOG	R	40, −72, −12	7.74	303
Occipital_Inf_L			L	−44, −72, −16	6.44	581
Fusiform_R	Fusiform Gyrus	FFG	R	38, −56, −22	6.89	938
Fusiform_L		(VWFA)	L	−42, −58, −18	5.86	230
Cerebellum						
Vermis_6	Cerebellum	(global maxima)	--	6, −62, −20	14.48	371
Cerebellum_4_5_R			R	16, −52, −22	12.26	732

Note: *p* < 0.05 FEW at peak level (uncorrected *p* = 6.2693 × 10^−6^, T = 4.4393).

**Figure A1:**
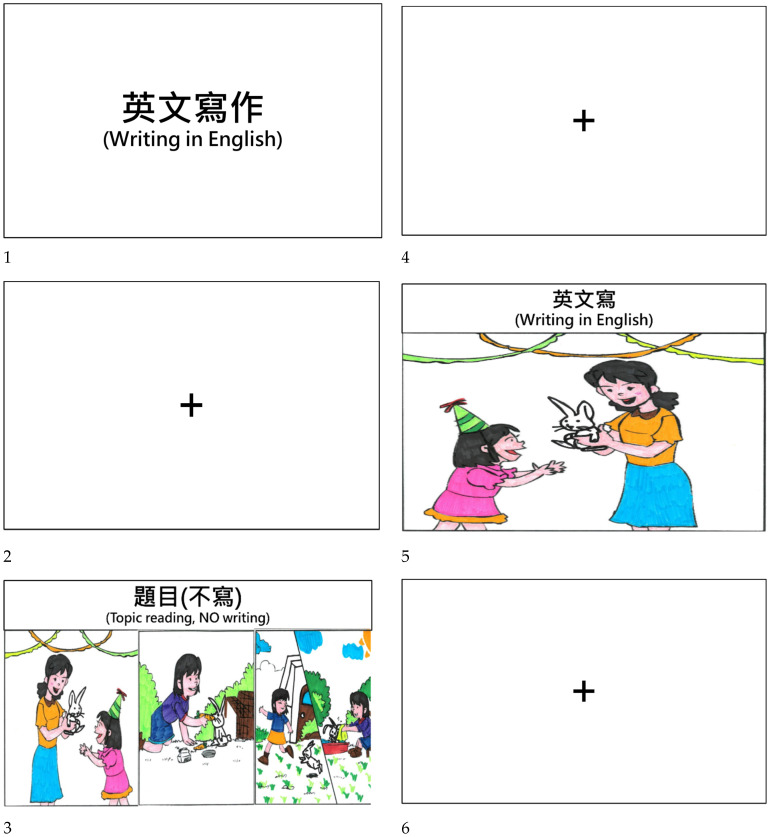
Task 3—Writing in Chinese; Task 4—Writing in English.

**Figure A2:**
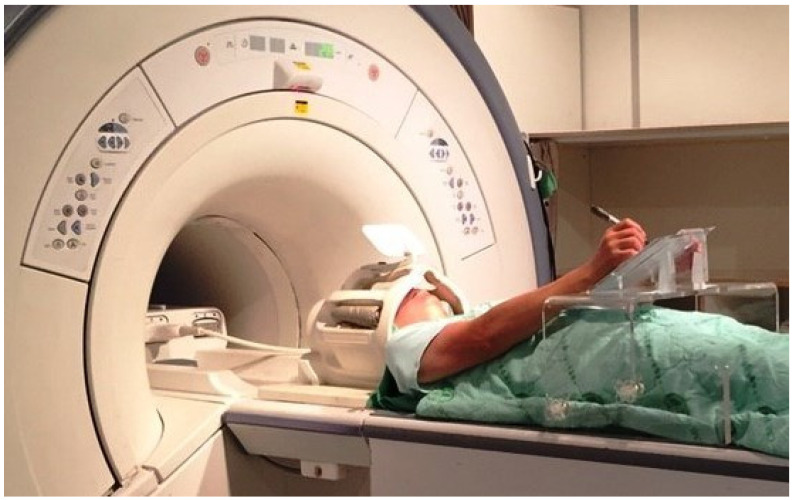
Layout of this experiment.

**Figure A3:**
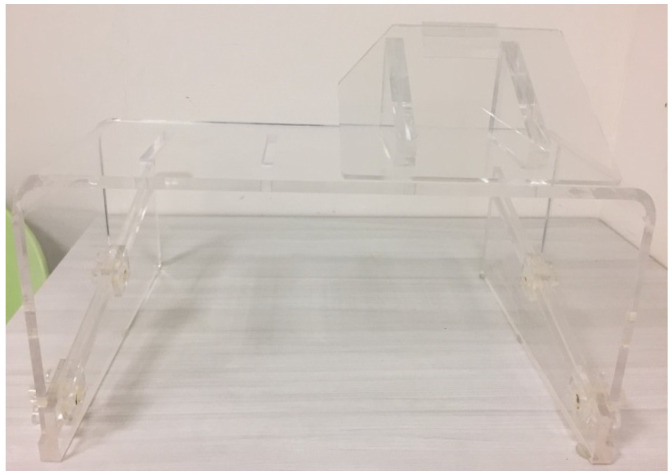
The writing pad.

**Figure A4:**
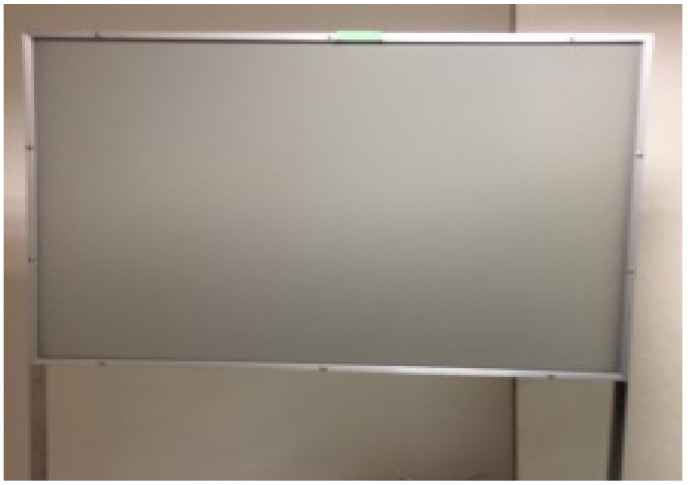
The projection screen.

## Data Availability

The data presented in this study are available on reasonable request from the corresponding author. The data are not publicly available because they are not fully ready yet.

## Appendix C. Further MANOVA Test

**Table A1:** Univariate Effects for “Group” Adjusted by Language Competence.

Dependent Variables		df/ df Error	*F*	Group	Means	99.9% Confidence Interval		Cohen’s d	Effect Size r
						Lower	Upper		
Task 1	Readability	1	0.01	Experiment	4.02	3.36	4.69	0.08	0.04
		50		Comparison	4.05	3.37	4.73		
	Speed	1	0.04	Experiment	4.03	3.19	4.74	0.07	0.03
		50		Comparison	3.97	3.25	4.83		
	Holistic	1	0.09	Experiment	4.10	3.43	4.64	0.21	0.10
		50		Comparison	4.17	3.61	4.85		
Task 2	Readability	1	1.64	Experiment	3.93	3.29	4.57	0.37	0.18
		50		Comparison	3.58	2.93	4.23		
	Speed	1	1.63	Experiment	4.03	3.42	4.65	0.38	0.19
		50		Comparison	3.70	3.07	4.32		
	Content	1	20.20 **	Experiment	3.73	3.21	4.35	1.37	0.57
		50		Comparison	2.67	2.03	3.20		
	Structure	1	13.94 **	Experiment	3.67	3.15	4.25	1.14	0.50
		50		Comparison	2.81	2.21	3.33		
	Grammar	1	3.29	Experiment	2.96	2.40	3.60	0.61	0.29
		50		Comparison	2.51	1.85	3.07		
	Vocabulary	1	10.40	Experiment	3.20	2.63	3.90	1.02	0.45
		50		Comparison	2.37	1.67	2.95		
	Punctuation	1	0.44	Experiment	3.54	2.93	4.26	0.28	0.14
		50		Comparison	3.36	2.63	3.99		
	Holistic	1	4.49	Experiment	3.48	2.95	4.09	0.70	0.33
		50		Comparison	2.97	2.35	3.51		
Task 3	Readability	1	1.53	Experiment	3.97	3.24	4.62	0.27	0.14
		50		Comparison	3.61	2.95	4.36		
	Speed	1	22.71 **	Experiment	4.06	3.41	4.67	1.31	0.55
		50		Comparison	2.78	2.16	3.45		
	Content	1	20.43 **	Experiment	3.92	3.28	4.58	1.33	0.55
		50		Comparison	2.67	1.99	3.32		
	Structure	1	20.61 **	Experiment	4.07	3.41	4.74	1.31	0.55
		50		Comparison	2.78	2.09	3.45		
	Grammar	1	26.06 **	Experiment	4.20	3.53	4.84	1.42	0.58
		50		Comparison	2.79	2.14	3.47		
	Vocabulary	1	42.83 **	Experiment	4.17	3.55	4.74	1.83	0.67
		50		Comparison	2.52	1.93	3.14		
	Punctuation	1	8.52	Experiment	4.22	3.65	4.80	0.85	0.39
		50		Comparison	3.51	2.92	4.09		
	Holistic	1	23.94 **	Experiment	4.13	3.54	4.68	1.36	0.56
		50		Comparison	2.94	2.37	3.54		
Task 4	Readability	1	3.70	Experiment	3.68	3.11	4.22	0.53	0.26
		50		Comparison	3.22	2.67	3.80		
	Speed	1	10.05	Experiment	3.89	3.37	4.48	0.98	0.44
		50		Comparison	3.16	2.55	3.68		
	Content	1	17.75 **	Experiment	3.45	2.88	4.08	1.27	0.54
		50		Comparison	2.38	1.74	2.96		
	Structure	1	11.46	Experiment	3.36	2.81	4.01	1.06	0.47
		50		Comparison	2.51	1.85	3.08		
	Grammar	1	2.25	Experiment	2.83	2.33	3.45	0.57	0.27
		50		Comparison	2.49	1.86	2.99		
	Vocabulary	1	8.35	Experiment	3.15	2.57	3.88	0.94	0.43
		50		Comparison	2.39	1.64	2.97		
	Punctuation	1	1.69	Experiment	3.42	2.81	4.15	0.48	0.23
		50		Comparison	3.06	2.32	3.68		
	Holistic	1	5.83	Experiment	3.33	2.79	3.95	0.78	0.36
		50		Comparison	2.74	2.11	3.29		

Note: ** *p* < 0.001.

## References

[1] Benko S.L. (2012). Scaffolding: An ongoing process to support adolescent writing development. J. Adolesc. Adult Lit..

[2] Schwieter J.W. (2010). Developing second language writing through scaffolding in the ZPD: A magazine project for an authentic audience. J. Coll. Teach. Learn..

[3] Karimpoor M., Churchill N.W., Tam F., Fischer C.E., Schweizer T.A., Graham S.J. (2018). Functional MRI of handwriting tasks: A study of healthy young adults interacting with a novel touch-sensitive tablet. Front. Hum. Neurosci..

[4] Hosseini S.H., Bruno J.L., Baker J.M., Gundran A., Harbott L.K., Gerdes J.C., Reiss A.L. (2017). Neural, physiological, and behavioral correlates of visuomotor cognitive load. Sci. Rep..

[5] Zhao H. (2018). New insights into the process of peer review for EFL writing: A process-oriented socio-cultural perspective. Learn. Instr..

[6] Paas F., Ayres P. (2014). Cognitive load theory: A broader view on the role of memory in learning and education. Educ. Psychol. Rev..

[7] Mourssi A. (2013). Theoretical and practical linguistic shifting from product/guided writing to process writing and recently to the innovated writing process approach in teaching writing for second/foreign language learners. Int. J. Acad. Res. Bus. Soc. Sci..

[8] Sweller J. (1994). Cognitive load theory, learning difficulty, and instructional design. Learn. Instr..

[9] Kalyuga S., Singh A.-M. (2016). Rethinking the boundaries of cognitive load theory in complex learning. Educ. Psychol. Rev..

[10] Vygotsky L. (1987). Zone of proximal development. Mind Soc. Dev. High. Psychol. Process..

[11] Cabell S.Q., Tortorelli L.S., Gerde H.K. (2013). How do I write…? Scaffolding preschoolers’ early writing skills. Read. Teach..

[12] Hyland K. (2007). Genre pedagogy: Language, literacy and L2 writing instruction. J. Second Lang. Writ..

[13] Rodd J.M., Vitello S., Woollams A.M., Adank P. (2015). Localising semantic and syntactic processing in spoken and written language comprehension: An Activation Likelihood Estimation meta-analysis. Brain Lang..

[14] Murphy E. (2020). The Oscillatory Nature of Language.

[15] Quiñones I., Molinaro N., Mancini S., Hernández-Cabrera J.A., Carreiras M. (2014). Where agreement merges with disagreement: fMRI evidence of subject–verb integration. NeuroImage.

[16] Mack J., Meltzer-Asscher A., Barbieri E., Thompson C. (2013). Neural correlates of processing passive sentences. Brain Sci..

[17] Wilson S.M., DeMarco A.T., Henry M.L., Gesierich B., Babiak M., Mandelli M.L., Miller B.L., Gorno-Tempini M.L. (2014). What role does the anterior temporal lobe play in sentence-level processing? Neural correlates of syntactic processing in semantic variant primary progressive aphasia. J. Cogn. Neurosci..

[18] Shetreet E., Friedmann N. (2014). The processing of different syntactic structures: fMRI investigation of the linguistic distinction between wh-movement and verb movement. J. Neurolinguist..

[19] Herrmann B., Obleser J., Kalberlah C., Haynes J.-D., Friederici A.D. (2012). Dissociable neural imprints of perception and grammar in auditory functional imaging. Hum. Brain Mapp..

[20] Flick G., Pylkkänen L. (2020). Isolating syntax in natural language: MEG evidence for an early contribution of left posterior temporal cortex. Cortex.

[21] Obleser J., Kotz S.A. (2010). Expectancy constraints in degraded speech modulate the language comprehension network. Cereb. Cortex.

[22] Ruff I., Blumstein S.E., Myers E.B., Hutchison E. (2008). Recruitment of anterior and posterior structures in lexical–semantic processing: An fMRI study comparing implicit and explicit tasks. Brain Lang..

[23] Snijders T.M., Vosse T., Kempen G., Van Berkum J.J.A., Petersson K.M., Hagoort P. (2009). Retrieval and unification of syntactic structure in sentence comprehension: An fMRI study using word-category ambiguity. Cereb. Cortex.

[24] Kambara T., Tsukiura T., Yokoyama S., Takahashi K., Shigemune Y., Miyamoto T., Takahashi D., Sato S., Kawashima R. (2013). Differential contributions of the inferior parietal and inferior frontal regions to the processing of grammatical and semantic relationships in wh-questions. Lang. Sci..

[25] Meltzer J.A., McArdle J.J., Schafer R.J., Braun A.R. (2010). Neural aspects of sentence comprehension: Syntactic complexity, reversibility, and reanalysis. Cereb. Cortex.

[26] Meyer L., Obleser J., Anwander A., Friederici A.D. (2012). Linking ordering in Broca’s area to storage in left temporo-parietal regions: The case of sentence processing. NeuroImage.

[27] Friederici A.D., Kotz S.A., Scott S.K., Obleser J. (2010). Disentangling syntax and intelligibility in auditory language comprehension. Hum. Brain Mapp..

[28] Lee D., Newman S.D. (2010). The effect of presentation paradigm on syntactic processing: An event-related fMRI study. Hum. Brain Mapp..

[29] Segaert K., Menenti L., Weber K., Petersson K.M., Hagoort P. (2011). Shared syntax in language production and language comprehension—an fMRI study. Cereb. Cortex.

[30] Planton S., Jucla M., Roux F.-E., Démonet J.-F. (2013). The “handwriting brain”: A meta-analysis of neuroimaging studies of motor versus orthographic processes. Cortex.

[31] Shah C., Erhard K., Ortheil H.-J., Kaza E., Kessler C., Lotze M. (2013). Neural correlates of creative writing: An fMRI Study. Hum. Brain Mapp..

[32] Wu J.R. (2012). GEPT and English language teaching and testing in Taiwan. Lang. Assess. Q..

[33] Lin S.H. (2020). Explanation of the Grading Standard for English Writing Ability in the 2020 College Entrance Examination.

[34] SPSS (2012). IBM SPSS Statistics Version 21.

[35] Friston K.J., Ashburner J.T., Kiebel S., Nichols T.E., Penny W.D. (2007). Statistical Parametric Mapping: The Analysis of Functional Brain Images.

[36] Lombardo M.V., Auyeung B., Holt R.J., Waldman J., Ruigrok A.N.V., Mooney N., Bullmore E.T., Baron-Cohen S., Kundu P. (2016). Improving effect size estimation and statistical power with multi-echo fMRI and its impact on understanding the neural systems supporting mentalizing. NeuroImage.

[37] Faul F., Erdfelder E., Buchner A., Lang A.-G. (2009). Statistical power analyses using G* Power 3.1: Tests for correlation and regression analyses. Behav. Res. Methods.

[38] Kawagoe T., Onoda K., Yamaguchi S. (2019). The neural correlates of “mind blanking”: When the mind goes away. Hum. Brain Mapp..

[39] Hoffstaedter F., Grefkes C., Caspers S., Roski C., Palomero-Gallagher N., Laird A.R., Fox P.T., Eickhoff S.B. (2014). The role of anterior midcingulate cortex in cognitive motor control: Evidence from functional connectivity analyses. Hum. Brain Mapp..

[40] Hervais-Adelman A., Egorova N., Golestani N. (2018). Beyond bilingualism: Multilingual experience correlates with caudate volume. Brain Struct. Funct..

